# Investigating Physicians’ Adoption of Telemedicine in Romania Using Technology Acceptance Model (TAM)

**DOI:** 10.3390/healthcare12151531

**Published:** 2024-08-01

**Authors:** Abigaela Bîlbîie, Andreea-Ionela Puiu, Viorel Mihăilă, Marin Burcea

**Affiliations:** 1 Faculty of Theoretical and Applied Economics, The Academy of Economic Studies, 010552 Bucharest, Romania; bilbiieabigaela@gmail.com; 2 Department of Applied Economics and Quantitative Analysis, Faculty of Business and Administration, University of Bucharest, 030018 Bucharest, Romania; 3 Department of Public Administration, Faculty of Business and Administration, University of Bucharest, 030018 Bucharest, Romania; viorel.mihaila@faa.unibuc.ro (V.M.); marin.burcea@faa.unibuc.ro (M.B.)

**Keywords:** telemedicine acceptance, physicians telemedicine acceptance, technology acceptance model, perceived usefulness, perceived ease of use

## Abstract

This study investigates Romanian physicians’ acceptance of telemedicine using the Technology Acceptance Model. We analyzed 1093 responses to an online survey distributed nationwide to physicians via email by the National Authority of Quality Management in Health, employing the partial least squares algorithm to estimate the relationship between the behavioral intention to adopt telemedicine and its potential determinants. Our findings reveal that the model accounts for 84.6% of the variance in behavioral intention to use telemedicine. Among the two constructs of the TAM model, perceived usefulness is a stronger predictor of behavioral intention than perceived ease of use. Additionally, subjective norms positively and significantly influence physicians’ intention to use telemedicine and their perception of its usefulness. Furthermore, perceived incentives and accessibility to medical records also positively impact the behavioral intention to use telemedicine.

## 1. Introduction

The emergence of COVID-19 brought public health back into focus, and there was a collective global effort to tackle this issue as effectively as possible [1]. A major driving force behind this worldwide endeavor was the widely recognized fact that public health is a vital and basic human right, as well as a significant factor that influences economic growth [2]. Healthy individuals exhibit higher productivity and enhanced physical and mental robustness, enabling them to work more efficiently and positively contributing to overall economic performance [3]. In addition to the emphasis on public health, the COVID-19 pandemic has compelled governments and international organizations to seriously consider the impact of this crisis on science, technology, and innovation [4]. As a result, healthcare stakeholders have had to acknowledge the importance of the digital transformation of the healthcare system [5] not only to address patients’ needs [6], but also “to improve healthcare industry’s efficiency, productivity and overall value” [7].

In a wide sense, digital transformation in healthcare encompasses all digital technologies and Internet-related adjustments that aim to improve health management procedures, including the development of new therapies and best practices [8]. The digitalization of the healthcare system focuses on the development of two main aspects of public health, namely patients’ experience and healthcare infrastructure [9]. The post-pandemic healthcare industry is confronted with the new “consumer of health care” who desires more integrated, effortless, and holistic healthcare services [10], along with an improved healthcare experience characterized by customization, comfort, and speed [8]. Regarding the impact of digitalization in healthcare infrastructure, Gjellebæk et al. [9] suggest that it could enhance employee productivity and the efficiency and effectiveness of health units and decrease their operational expenses.

In the sphere of digital health, telemedicine is a component that makes “use of information and communication technologies to improve patient outcomes by increasing access to care and medical information” [11]. More precisely, telemedicine involves the provision of healthcare services from a distance. During the COVID-19 pandemic, telemedicine was the only option for minimizing the spread of the disease by remotely monitoring and treating patients, including those infected with COVID-19 as well as those with other chronic conditions. From a medical standpoint, telemedicine has enabled doctors to remotely monitor patients, facilitating the early detection of COVID-19 and timely treatment of infected individuals. It has also ensured the uninterrupted care of vulnerable patients with other medical conditions [12]. 

This study aims to investigate the level of telemedicine adoption among physicians in Romania and identify the factors or drivers that influence their acceptance of this technology.

For this study, we adapted the third version of the Technology Acceptance Model (TAM3) to apply to telemedicine services. We also introduce additional constructs, namely clinical factors, and regulatory factors, to gain a better understanding of how physicians perceive telemedicine. Using partial least squares path modeling, conducted in WarpPLS, version 8.0, the study not only examines the statistical relevance of the predictive factors proposed by an adapted version of TAM3 but also identifies the most appropriate determinants that can be used to enhance the intention of physicians to adopt telemedicine services.

The rest of the paper is organized as follows: Section 2 of this scientific article aims to provide a comprehensive understanding of the existing research landscape, identify gaps, and establish the theoretical foundation for the current study, encompassing both the theoretical framework and hypotheses development. Moving into Section 3, detailed information is presented regarding the acquisition of data, the chosen metrics and instruments, as well as the research design and statistical techniques applied, while Section 4 presents the results. Section 5 critically interprets and contextualizes the findings in relation to the theoretical framework and the prior literature. 

## 2. Literature Review

### 2.1. Telemedicine in the Healthcare Sector

During this challenging period, health specialists had to recognize the significance of telemedicine in healthcare, not only during pandemics or epidemics but also in the routine delivery of healthcare services. Furthermore, the public health crisis precipitated by the pandemic has generated heightened interest from patients and an increased demand for healthcare delivery through virtual means. To illustrate this impact more comprehensively, we can look at pertinent statistics from the United States where, over the period spanning 2019 to 2022, there was a remarkable 63-fold increase in the number of virtual medical consultations [7,13].

The primary factors that have led to the increased attention towards telemedicine among healthcare providers include its capacity to enhance access to healthcare [14], increase convenience [15], reduce treatment costs [16], and decrease hospital admissions [17] or readmissions, especially in the Accident and Emergency department, where a patient visit could be much more expensive [18,19]. Telemedicine can enhance hospital efficiency by automating routine tasks using AI, which allows medical personnel to allocate more time to other aspects of their work [20]. A 2019 study suggests that the National Health Service (NHS) could reduce staff costs by GBP 12.5 billion annually through automation, while the social care industry could save GBP 5.9 billion [21].

Furthermore, telemedicine has the potential to improve patient–physician interactions and reduce misunderstandings, which account for around 80% of severe medical injuries [7]. The healthcare sector generates vast amounts of data annually, including clinical records, medical imagery, genomic information, and health behavior data from patients, organizations, and monitoring devices [22]. Therefore, using big data in healthcare can reduce medication or medical mistakes, improve comprehension of patient trends, provide accurate staffing predictions, examinate real-time alerts, and develop preventive care strategies [7].

The medical fields where telemedicine is used the most include radiology [23], psychiatry, mental health [24], and cardiology, while the lowest usage of telemedicine is recorded in allergology/immunology, gastroenterology, and obstetrics/gynecology [25]. Also, emergency physicians, pathologists, and radiologists are among the healthcare professionals who use telemedicine the most to communicate with other healthcare providers [25].

In Romania, before 2020, telemedicine was not a priority for the Romanian government or public health system, and existing pilot projects were underdeveloped. However, the pandemic prompted authorities to establish a legal framework to promote telemedicine [26,27]. Based on a 2021 study, Romania has the highest proportion of telemedicine enthusiasts among E.U. countries (69%), yet only 28% believe that telemedicine functions effectively in the country [28].

Regarding physicians’ acceptance of telemedicine services, previous research has indicated that medical professionals from developing countries are less inclined to adopt telemedicine [29]. In a particular county in Romania, a cross-sectional study conducted in 2020 revealed that 61% of surveyed physicians found telemedicine consultations more time-consuming than in-person visits, and 64% were concerned about the reliability of telemedicine diagnostics [30].

### 2.2. Technology Acceptance Model

The Technology Acceptance Model (TAM) was initially proposed by Davis [31] in 1989 as part of his doctoral research, drawing upon the foundations of the Theory of Reasoned Action [32]. The basic technology acceptance model framework assumes that people’s behavioral intention to use information technology (IT) is influenced by two personal beliefs, namely perceived usefulness (PU) and perceived ease of use (PEOU), along with the attitude towards technology adoption. Perceived usefulness and perceived ease of use also play a role in how other external factors affect the intention to use IT [33]. According to [31], perceived usefulness refers to how much an individual believes that using IT will improve their job performance, while perceived ease of use is the extent to which an individual believes that using IT will be effortless or without much effort.

Venkatesh and Davis [34] expanded the TAM into TAM2, identifying and conceptualizing determinants affecting perceived usefulness such as subjective norm, image, job relevance, output quality, result demonstrability, and perceived ease of use, with experience and voluntariness as moderators. Further, Venkatesh [35] formulated a model that explores the determinants influencing perceived ease of use, based on the anchoring and adjustment framing of human decision-making processes. In this model, computer self-efficacy, computer anxiety, computer playfulness, and perceived external control were explored as anchors, while perceived enjoyment and objective usability were framed as adjustments. TAM3 represents the integrated model of TAM2 and the model of determinants of perceived ease of use.

The extensive popularity of the TAM model and its extensions relies on its ability to explain and predict human behavior in the acceptance of technology across various disciplines, contexts, and geographical locations. This is achieved through the concise structure of perceived usefulness and perceived ease of use, as well as the determinants that directly impact these constructs.

### 2.3. Hypotheses Development

The TAM and its extensions have been widely used to explain people’s behaviors in an Information System and technology acceptance [36]. For example, TAM3 has been successfully applied to explain a variety of behavioral intentions, such as the acceptance of technology in the construction industry [37], digital banking channel acceptance [38], the social network impact on learning [39], the behavioral intention to use a smart village [40], m-commerce adoption [41], and many more. TAM3 is also used to investigate different areas of the healthcare industry, such as understanding key factors influencing the adoption of hospital electronic health records [42], identifying factors that predict the likelihood to adopt tele-neurorehabilitation [43], determining factors influencing nurses’ acceptance of a hospital information system [44], or the factors influencing the acceptance of teleorthodontic technology [45]. Over time, the TAM framework has been extensively used to analyze the perceptions and adoption of health information technology by healthcare providers [46,47], especially in health services such as telemedicine, electronic health records, and mobile health applications [48].

In this research, we adapt to telemedicine services the third iteration of the technology acceptance model (TAM3) introduced by Venkatesh and Bala [33]. We specifically choose this model due to its inclusive methodology in examining the factors that influence perceived ease of use, and that is because when individuals perceive a technology as being user-friendly, it enhances their confidence and competence in adopting and using the technology effectively [49].

In addition to TAM3, our study includes two constructs derived from clinical factors identified in previous studies [50,51,52], namely accessibility to medical records and accessibility to patients.

Based on Venkatesh and Bala’s [33] findings, the original TAM3 predicts behavioral intention over various periods and models with 40% to 53% accuracy, with perceived usefulness and perceived ease of use as significant predictors of behavioral intention—this finding is also supported by various other researchers [53,54,55]. Among these two predictors, perceived usefulness has a stronger predictive power than perceived ease of use [33,56,57]. Additionally, perceived ease of use not only influences behavioral intention but also plays a role in determining perceived usefulness. This is because a system that requires less effort to use can enhance people’s belief in its usefulness [34]. Based on these considerations, it is also assumed that the following research hypotheses hold true in this study case too:

**H1.**  *Perceived usefulness has a positive influence on the behavioral intention to use telemedicine*.

**H2.**  *Perceived ease of use has a positive influence on the behavioral intention to use telemedicine*.

**H3.**  *Perceived ease of use has a positive influence on perceived usefulness*.

In addition to examining perceived usefulness and perceived ease of use as main predictors of the behavioral intention to use telemedicine, our study also incorporates the construct of perceived incentives. This construct, classified as a regulatory factor, refers to the “extent to which an individual believes that the provision of telemedicine services would be rewarded with financial support or compensation for medical expenses” [58] and can directly influence the behavioral intention towards telemedicine, either positively or negatively. A lack of clear regulations or incentives often hinders telemedicine adoption [33,51,59], while high incentives increase healthcare professionals’ willingness to embrace a new technology [58]. Thus, to better understand the role of perceived incentives among Romanian physicians, we set the following hypothesis:

**H4.**  *Perceived incentives positively influence the behavioral intention to use telemedicine*.

Perceived usefulness can be influenced by different determinants, including subjective norms [34], and clinical factors like accessibility of medical records and accessibility of patients [51]. Subjective norms represent the extent to which individuals perform a certain behavior to obtain approval from a referent group and are expected to positively influence perceived usefulness and behavioral intention [33]. Previous studies show that the accessibility of medical records and patients enhances physician satisfaction, speeds up care delivery, reduces healthcare service time, and lowers costs [50,52]. Rho et al. [51] found that the accessibility of patients affects both perceived usefulness and the behavioral intention to use telemedicine. In this study, we want to examine whether the accessibility of medical records can directly influence the behavioral intention to use telemedicine services. We therefore set the following hypotheses:

**H5.**  *Subjective norms have a positive influence on perceived usefulness*.

**H6.**  *Accessibility of medical records has a positive influence on perceived usefulness*.

**H7.**  *Accessibility of patients has a positive influence on perceived usefulness*.

**H8.**  *Subjective norms have a positive influence on behavioral intention*.

**H9.**  *Accessibility of medical records has a positive influence on behavioral intention*.

**H10.**  *Accessibility of patients has a positive influence on behavioral intention*.

The factors contributing to perceived ease of use, as proposed by Venkatesh [35], based on the anchoring framing of human decision-making, include computer self-efficacy, computer anxiety, and perceptions of external control. These factors are based on the idea that individuals’ perceptions of how easy technology is to use are anchored to their past computer experiences. Computer self-efficacy refers to one’s belief in their ability to effectively use a system, such as telemedicine [33]. Conversely, computer anxiety involves fear of the unknown, frustration, embarrassment, failure, or disappointment, which can lead to avoidance of using the technology [60]. Perceptions of external control relate to the belief that organizational and technical resources will provide adequate support for using the system [33,61]. Taking this into account, we set the research hypotheses outlined below:

**H11.**  *Computer self-efficacy has a positive influence on perceived ease of use*.

**H12.**  *Perceived external control has a positive influence on perceived ease of use*.

**H13.**  *Computer anxiety has a negative influence on perceived ease of use*.

**Figure 1:**
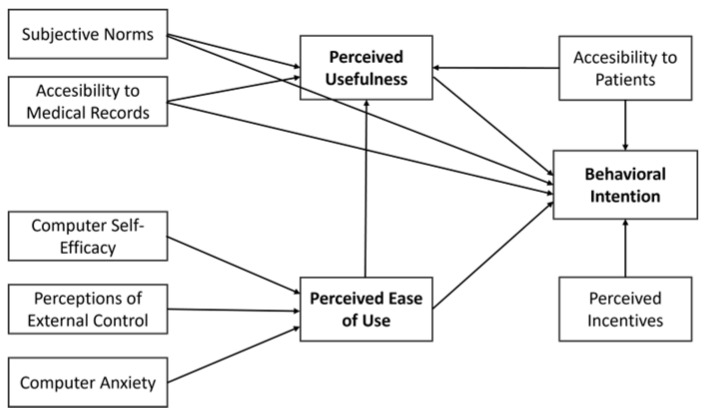
The research model.

Understanding these determinants and their influence on Romanian physicians’ adoption of telemedicine can help policymakers pinpoint areas for improvement, boosting telemedicine use and productivity. Moreover, integrating TAM3 with other factors can offer a more detailed explanation of physicians’ acceptance and use of telemedicine services [51]. The conceptual model is presented in Figure 1. 

## 3. Materials and Methods

### 3.1. Data

Data were gathered from 8th February to 26th February 2023, through a self-reported survey created on Google Forms. The survey was distributed to physicians nationwide via email by the National Authority of Quality Management in Health (ANMCS). The privacy and anonymity of the participants were ensured during the data collection process, and no ethical issues were identified, following established practices in socioeconomic research. Out of 5200 distinct emails sent to different physicians throughout Romania by the ANMCS, 1093 completed the questionnaire. Participants did not receive reminders to complete the questionnaire. The survey response rate was 21.02%. All variables’ questions were measured using a 7-point Likert scale because it supplies a better accuracy estimate of participants’ evaluation, compared to a 5-point Likert scale, and is more suitable for electronically distributed and unsupervised usability questionnaires [62,63].

### 3.2. Measurement

This study aims to investigate the determinants that influence the acceptance of telemedicine among physicians in Romania and the impact of these determinants on their intention to use this service. By comprehending these aspects, we can gain a clearer understanding of the feasibility of implementing telemedicine in Romania and its potential to enhance efficiency and productivity in public healthcare systems.

In this research, we adapted the TAM3 framework to suit the context of telemedicine services and adjusted the variables employed. Among the factors measuring TAM3 dimensions, we excluded one moderator, namely experience. We proceeded this way because, in the TAM3 model [33], ‘experience’ is mentioned as a moderator but is not listed as a variable. Instead, the model includes a variable called ‘use’. Our study uses a similar question under the variable ‘Telemed’, asking if respondents have provided telemedicine services in the past year. However, this variable does not show statistical significance in our results. Additionally, we excluded image construct, which influences perceived usefulness, as well as computer playfulness, a variable that affects perceived ease of use. We also excluded the constructs of perceived enjoyment and objective usability, comprising related adjustment. Although we collected data on variables such as voluntariness, job relevance, output quality, and result demonstrability, we decided not to include them in the analysis due to identified endogeneity issues with behavioral intention. The questionnaire employed in this study incorporates items drawn from the work of Rho et al. [51] for assessing behavioral intention to use, perceived usefulness, perceived ease of use, perceived incentives, accessibility of medical records, and accessibility of patients. Additionally, items proposed by Venkatesh and Bala [33] are employed to measure computer self-efficacy, perceptions of external control, computer anxiety, and subjective norms. The questionnaire is available for reference in Appendix C. 

### 3.3. Method

To assess the extent to which the predictors of the TAM, along with clinical and regulatory factors, account for variability in the outcome, we employed the partial least squares (PLS) algorithm [64]. The PLS algorithm consists of two main components: the inner model, which examines the connections between the latent variables within the PLS model, and the outer model, which explores the relationship between the latent variables and the observed variables in the PLS model. To conduct the analysis, we used WarpPLS, version 8.0.

## 4. Results

Our study encompasses 300 hospitals, predominantly located in urban areas, out of a total of 704 hospitals nationwide. Our sample consists of 1093 respondents (759 women and 334 men) with an average age of 46 years old. The largest age group within our sample, accounting for 33.4% of respondents, falls within the 40 to 49-year-old range. A total of 89.7% of the respondents have their provenience in urban areas. According to the national data provided by the National Institute of Statistics (INSSE) in their 2022 report [65], Romania has 71,293 physicians, with 92.3% practicing in urban areas and 7.7% in rural areas. Among these physicians, 70.8% are female. Subsequently, these comparisons validate the representativeness of our sample at the national level.

Also, 56.9% of them are primary care physicians, which can explain why around 69% of them have an income above RON 10,000 (EUR ~2000). As per the updated law 153/2017 in 2023, the base salary for a primary care physician is approximately RON 15,567 (EUR ~3100) [66]. A detailed description of our sample is available in Table 1. 

### 4.1. The Measurement (Outer) Model

Table 2 presents the measurement reliability of each construct used in the study. The composite reliability values range between 0.866 and 0.971, exceeding the recommended threshold of 0.70 [67]. Moreover, the Cronbach’s alpha values for most variables are higher than 0.80 indicating a high level of internal consistency, except for the perceived incentives variable which has a Cronbach’s alpha of 0.768 However, this value is still above the threshold of 0.70 [68], which is considered acceptable in many research situations [67]. Additionally, Table 2 indicates that all variables have an average variance extracted (AVE) greater than 0.50 [69], indicating strong convergent validity. The composite reliability, Cronbach’s alpha values, and AVE results suggest that the variables employed in our study possess both reliable and high-quality measurement questions. 

Table 3 demonstrates that the measurement exhibits discriminant validity, as indicated by the higher values on the diagonal compared to the corresponding off-diagonal values. While only a few off-diagonal correlations slightly exceed the recommended threshold of 0.8 [70], it is important to note that discriminant validity, as assessed by the cross-loadings, is influenced by various factors. These factors include the correlations between constructs, the measurement process, and the characteristics of the sample. Each of these factors has distinct implications for determining which values should be considered high and potentially problematic [71].

The loadings of all reflective items fall within the range of 0.753 to 0.968, surpassing the minimum threshold of 0.7 [72,73,74], which indicates that the constructs capture the variance of the considered items. Additionally, the off-diagonal values for each set of measurement items are lower than the corresponding diagonal values, indicating good discriminant validity among the indicators. The combined loadings and cross-loadings of the indicator items for the latent construct are available in Appendix B.

### 4.2. The Inner Model

Appendix A presents the estimated coefficients of the model, while Appendix B shows the effect sizes for direct, indirect, and total effects. The analysis indicates that the model explains 84.6% of the behavioral intention to use telemedicine, as well as 71.5% of the variance in perceived usefulness and 72.7% of the variance in perceived ease of use. The overall model has a Tenenhaus Goodness of Fit (GoF) value of 0.82, exceeding the recommended threshold of 0.36 In the absence of other studies using a model similar to ours for comparison, we can also highlight the good performance of the TAM3 model developed by Venkatesh and Bala [33], which served as the foundation for our model, along with the model of Rho et al. [51], from which we incorporated two variables: accessibility of medical records and accessibility of patients. This can serve as further evidence that our model effectively captures the underlying patterns and relationships present in the data. Also, the model demonstrates an absence of endogeneity, signifying that the variables are not subjected to biased and inefficient estimates in statistical analyses. We checked for multicollinearity, and the average block VIF (AVIF), an index that tends to increase if new latent variables are added to the model and they overlap in meaning with existing latent variables, is 1.998, which is below the recommended threshold of 3.3 [75,76,77]. Therefore, full collinearity was not identified. 

#### 4.2.1. Predictors of Behavioral Intention

Perceived usefulness (β = 0.179, *p* < 0.001) and perceived ease of use (β = 0.098, *p* < 0.001) demonstrate a significant positive association with the intention to use telemedicine, supporting H1 and H2. In terms of predictive power, perceived usefulness exhibits a higher effect size (0.146) compared to perceived ease of use (0.073), indicating that perceived usefulness has a stronger impact on the behavioral intention to use telemedicine than perceived ease of use. Additionally, perceived ease of use positively and significantly influences perceived usefulness (β = 0.283, *p* < 0.001), providing support for H3.

Perceived incentives, our regulatory factor in the study, positively influence behavioral intention (β = 0.120, *p* < 0.001), supporting H4. Additionally, subjective norms (β = 0.303, *p* < 0.001) and accessibility of patients (β = 0.278, *p* < 0.001) demonstrate a positive predictive effect on the behavioral intention to use telemedicine. Hence, H8 and H9 are supported. As we expected, the accessibility of medical records (β = 0.254, *p* < 0.001) also emerged as a variable that positively influences the behavioral intention to adopt telemedicine services, supporting the H10 hypothesis. 

Out of all the variables that influence the behavioral intention to use telemedicine services, subjective norms have the greatest effect size (0.243) followed by accessibility of patients (0.228) and accessibility of medical records (0.204).

#### 4.2.2. Predictors of Perceived Usefulness

Subjective norms (β = 0.287, *p* < 0.001), accessibility of medical records (β = 0.255, *p* < 0.001), as well as accessibility of patients (β = 0.142, *p* < 0.001), significantly predict perceived usefulness, exerting a positive influence on this construct. Thus, H5, H6, and H7 are supported. Of all these variables, subjective norms and perceived ease of use (both with an effect size of 0.211) demonstrate a stronger predictive ability for perceived usefulness compared to accessibility of medical records (effect size = 0.191) or accessibility of patients (effect size = 0.103).

#### 4.2.3. Predictors of Perceived Ease of Use 

Computer self-efficacy (β = 0.280, *p* < 0.001) and perceptions of external control (β = 0.613, *p* < 0.001) are positively and significantly associated with perceived ease of use, while computer anxiety (β = −0.078, *p* = 0.005) is negatively and significantly correlated with perceived ease of use (β = −0.078, *p* = 0.005). Therefore, we can confirm support for H11, H12, and H13. Among the three predictors of perceived ease of use, perceptions of external control exhibit the highest effect size (0.505), indicating that it has a stronger predictive power compared to the other two predictors.

Regarding age (β = 0.007, *p* = 0.406), previous use of telemedicine (β = 0.033, *p* = 0.135), and gender (β = −0.005, *p* =0.430), their influences on behavioral intention are positive and negative, respectively. However, none of these variables yield statistically significant effects on the intention to use telemedicine.

## 5. Discussion

We explored the factors influencing Romanian physicians’ intention to use telemedicine, using the third version of the Technology Acceptance Model (TAM) as our theoretical framework, with clinical and regulatory factors as additional variables. Our model explains 84.6% of the behavioral intention to use telemedicine, higher than the 53% identified in the work of Venkatesh and Bala [33]. Similarly, perceived usefulness (70.7% vs. 67%) and perceived ease of use (72.7% vs. 52%) were also higher than in the work of Venkatesh and Bala [33].

Our findings show that perceived usefulness and perceived ease of use, key variables in the Technology Acceptance Model (TAM), significantly and positively impact physicians’ intention to use telemedicine, with perceived usefulness being the stronger predictor. This result is supported by previous studies [33,51,78]. Additionally, a study carried out by Kohnke [79], focusing on telehealth use by patients, reached a similar conclusion. They found that both perceived usefulness and perceived ease of use were important factors in predicting the intention to use telehealth services. Between the two, the ease-of-use factor plays a more significant role in determining the intention to use telemedicine. Physicians, particularly those with prior experience in using medical technologies, hold a stronger belief in the usefulness of various e-health technologies, perceiving their advantages to outweigh any potential challenges or drawbacks [80].

Furthermore, our findings indicate that subjective norms play a positive and significant role in shaping physicians’ intention to use telemedicine, but also in the perceived usefulness of this technology. According to Venkatesh and Bala [33], subjective norms played a key role in predicting perceived usefulness and their impact on behavioral intention was influenced by factors such as experience and voluntariness. As individuals gained more experience, particularly in a voluntary setting, the influence of subjective norms became less significant. A study conducted in Senegal [81] also identified subjective norms as significant factors influencing physicians’ intentions to incorporate telemedicine into their professional practices. Specifically, their behavioral intention to adopt telemedicine is greatly influenced by the thoughts and actions of their reference group, such as family, friends, colleagues, and other significant ones [82].

However, in another study on Iranian medical students, subjective norms were not statistically significant in influencing students’ intention to use mobile health applications [83]. Also, in the study of Cohen et al. [84] on a South African population concerning electronic prescribing, subjective norms (or social influence) had a low impact on the usage of this technology. Those inconsistent findings can be interpreted considering Lee and Wan’s [85] perspective, which proposes that the varying results concerning the impact of subjective norms on technology adoption may be influenced by the individuals’ cultural context. Specifically, the distinction between individualistic and collectivist cultures may play a role in shaping the influence of subjective norms on technology adoption.

Individuals in individualistic societies pay less attention to the opinions of those around them, while those in collectivist societies value the opinions of their reference groups. By using a country comparison tool, we can observe that both Romania and Senegal (where subjective norms significantly influenced the behavioral intention to use technology) have scores of 30 and 25, respectively, on the Individualism dimension. This suggests that both countries are collectivist societies that rely on social networks for their identity. Conversely, South Africa (where subjective norms did not significantly impact intention to use) records a score of 65 on Individualism, indicating that South African individuals may have lower interpersonal connections with individuals outside their core “family” group [86,87]. Thus, it is essential to consider the various social and cultural contexts when interpreting the impact of subjective norms on the intention to use telemedicine. 

The accessibility of medical records significantly influences Romanian physicians’ inclination to adopt telemedicine and its perceived usefulness. This is primarily because Romania lacks a well-designed database capable of providing comprehensive medical data about patients. The Romanian healthcare system is marked by a lack of integration between different health sectors and a lack of progress in ensuring uninterrupted and interconnected healthcare services [88], leading to a higher risk of medical errors [89].

In 2014, the Romanian National Health Insurance House launched the project “The Patient’s Electronic Health Record” to consolidate all patient’s medical records, including consultations, referrals, analyses, prescriptions, observation sheets, and medical history, into a single platform [90]. However, the implementation of the electronic file was rushed and poorly prepared, presenting serious issues regarding protection of personal data of a medical nature contained in the Electronic Health Record, as the Ombudsman signaled to the Constitutional Court of Romania [91]. Consequently, shortly after its launch, the “Patient’s Electronic Health Record” encountered significant functionality issues for three and a half years, mostly due to data security vulnerabilities [92]. 

An updated version of The Patient’s Electronic Health Record was introduced in November 2021, but awareness among doctors and patients was low. Currently, 27,219 doctors from over 12,261 medical units (approximately 40% of Romania’s doctors) have submitted data [93], and only 0.07% of patients have accessed the medical information stored in these records. Most submissions within the Electronic Health Record come from family doctors, while very few hospitals share this information with the National Health Insurance House. The reason behind this issue is the lack of connectivity between the software systems used by medical units and those employed by the National Health Insurance House [94].

The accessibility of patients, the other clinical factor observed in our research, also exerts a significant positive effect on both behavioral intention and the perceived usefulness of using telemedicine. According to a European Commission’s report from 2022, Romania faces considerable healthcare accessibility challenges due to staff shortages, underdeveloped primary care, and high unmet needs (4.4%, one of the highest in EU). Additionally, the country experiences lower rates of consultations in primary and ambulatory care compared to the EU average. Despite producing many medical graduates, the healthcare system faces significant staff shortages and distribution imbalances due to workforce emigration, with 12% of doctors and 11% of nurses trained in Romania working elsewhere in the EU [95]. Romania has significantly limited healthcare access in rural areas, and this issue is further exacerbated by differences in demographic coverage (e.g., the Roma population and homeless people have very low access to healthcare) [88]. Therefore, healthcare professionals, particularly physicians who are deeply engaged in public health, experienced the greatest difficulties in delivering healthcare services to these vulnerable populations. As a result, telemedicine emerges as a potential solution that could enhance healthcare accessibility for patients who face obstacles in attending in-person medical consultations.

In this study, we also observed three determinants that previous studies have indicated could influence perceived ease of use [33,35]: perceptions of external control, computer self-efficacy, and computer anxiety. Our study reinforces that these factors significantly influence the extent to which a technology is perceived as being easy to use.

Perceptions of external control positively influence perceived ease of use and exert the highest influence on perceived ease of use, followed by computer self-efficacy. Perceptions of external control positively influence perceived ease of use and exert the highest influence on perceived ease of use, followed by computer self-efficacy. Healthcare technologies, encompassing various systems from health data management to diagnosis and care delivery, possess unique complexities compared to other technological domains. Understanding the technological landscape and user (i.e., physicians, medical staff, etc.) dynamics, along with their perspectives and concerns about these technologies, is essential for proposing context-specific solutions tailored to healthcare systems. Moreover, the integration of new health technology in healthcare requires adequate guidance to minimize complexity in its usage.

Comparing our results with previous findings, we observed that in the study conducted by Rho et al. [51] on South Korean physicians, computer self-efficacy was the dominant predictor of telemedicine acceptance. However, both constructs, as proposed by Venkatesh [35], represent different dimensions of control, which refers to “the individual’s perception of the availability of knowledge, resources, and opportunities required to perform the specific behavior”. While control has traditionally been seen as a unidimensional concept in research, Ajzen [96] revolutionized its conceptualization by introducing a bi-dimensional approach: internal control (i.e., knowledge/self-efficacy) and external control (i.e., environment). In the context of technology usage, internal control is represented by computer self-efficacy, whereas external control corresponds to perceptions of external control [35]. As the same study suggests, considering the broad conceptualization of perceived ease of use, we might also expect that people will incorporate both internal and external dimensions of control into their perceptions of external control.

Computer anxiety has a notable detrimental impact on technology usage, which is consistent with earlier research conducted by [33,35,97]. This implies that individuals with higher levels of technology anxiety are less likely to have the intention to use any form of technology.

Conversely, it appears that perceived incentives significantly and positively impact the adoption of telemedicine, consistent with the studies conducted in [51,80]. In addition, research conducted on Nigerian physicians by Adenuga et al. [98] revealed that financial incentives were influential in their propensity to use telemedicine services. Similarly, healthcare professionals from Morocco, as studied by Mohammed et al. [58], expressed that “appropriate support from governmental bodies, financial support for institutions that adopt this technology, and reimbursement for procedures conducted through the digital platform” had the potential to increase telemedicine usage. The significant role of incentives in healthcare was also highlighted by Glaser [99], who found low technology adoption in institutions with insufficient or nonexistent incentives. Additionally, Chaix-Couturier et al. [100] discovered that the inclusion of financial incentives led to an increase in night calls.

The minimal or non-significant effects of the control variables “age” and “gender” can be attributed to the fact that physicians, regardless of age and gender, receive professional training and ongoing education that often includes exposure to new technologies and practices, enhancing their confidence and willingness to adopt new technologies [61,101]. Thus, their acceptance of telemedicine might be more influenced by professional, institutional, or practical considerations than by their demographic characteristics, as this study’s findings have already suggested above.

## 6. Limitations and Future Directions

There is no doubt that this study has provided valuable insights regarding telemedicine acceptance by physicians in Romania, since the research on telemedicine acceptance in Romania is almost nonexistent, and therefore our results cannot be judged against local theoretical backgrounds or previous findings. However, we must consider the limitations and future directions of this study, which will be briefly discussed below. 

First, most of the study participants (~89.7%) were from urban areas. This is because the study was based on the database of the National Authority of Quality Management in Health, which primarily includes physicians from urban medical units. However, having a study sample predominantly composed of physicians from urban areas can impact the generalizability of the study’s findings, since they may not accurately represent the attitudes, behaviors, and challenges faced by physicians from rural areas. Moreover, the healthcare delivery and patient demographics can vary significantly between urban and rural areas. This is particularly relevant given that rural areas often have a higher proportion of older patients compared to urban areas [102]. These older patients may find technology too complicated to understand [103], which can influence the adoption of telemedicine among both patients and physicians. Taking these aspects into account, future research might consider a deliberate sampling from diverse geographic locations, ensuring a more representative reflection of physicians across urban and rural settings.

Second, telemedicine is currently underdeveloped in Romania [26] and this may be a limitation of this study in the way that physicians may have limited exposure to telemedicine practices in the public health system, impacting their perceptions and attitudes and making it challenging to draw comprehensive conclusions about telemedicine acceptance. In addition to the previously mentioned National Electronic Health Record, which has significant shortcomings, the Ministry of Health initiated a bid in 2023 [104] to procure comprehensive telemedicine solutions, intended to outfit 131 Emergency Reception Units. However, this initiative represents a relatively small step forward and is insufficient to establish an integrated telemedicine system in Romania. Furthermore, the insufficient overall technological assistance and resources for various digital healthcare solutions implemented in Romania (i.e., the Electronic Health Record) thus far may foster uncertainty, resistance, or skepticism among physicians regarding the adoption of telemedicine. Consequently, these characteristics pose limitations on the generalizability of findings to other countries or contexts. Future research in regions with diverse telemedicine implementations can provide a more comprehensive understanding of the factors influencing telemedicine acceptance among physicians.

Third, the model employed in this study did not measure constructs associated with trust, data protection concerns, and cybersecurity within the telemedicine context. Evaluating physicians’ levels of trust in telemedicine has the potential to yield a more comprehensive understanding of the psychological and attitudinal factors that either contribute to or impede its adoption. Furthermore, trust and security considerations are often interconnected with policy and implementation decisions in telemedicine, providing a more nuanced comprehension of the factors influencing physicians’ acceptance of telemedicine and facilitating the development of strategies to address potential barriers. Therefore, to enhance the study’s robustness and applicability, future research should consider integrating measurements for trust in telemedicine and variables related to personal data and cybersecurity.

## 7. Conclusions

This research is timely, with a consistent practical footprint. The Romanian legislation on telemedicine services has been steadily updated from 2020 onward, with EMERGENCY ORDINANCE no. 196 of 18 November 2020 for the amendment and completion of Law no. 95/2006 regarding health reform, followed by the DECISION no. 1133 of 14 September 2022 regarding the approval of the Methodological Norms for the implementation of the provisions of the Government Emergency Ordinance no. 196/2020 for the amendment and completion of Law no. 95/2006 on health reform. Moreover, within Romania’s recovery and resilience plan, funded by the EU till 2026, the Romanian government will fund the implementation of e-health and telemedicine systems within the digital transformation pillar. Some other initiatives have also been supported by the EU in the previous years, such as the project POSCCE 49472—“Increasing the quality of the medical act in rural areas by implementing a Telemedicine Information System”—financed by The European Regional Development Fund under the Increase of Economic Competitiveness, Sectorial Operational Program 2007–2013.

While the policy perspectives have started to be better articulated, as always, in real life, things are a bit more complicated. For the researched area of medical professionals working in urban areas in hospital settings, the adoption of telemedicine services as a routine act is still in its infancy. Uncovering the intricacies of medical professionals’ decision-making processes when contemplating telemedicine services will help the authorities speed up the process.

Telemedicine is a promising field that offers numerous opportunities for innovation and investment, having the potential to enhance the quality of healthcare services through technological advancements in the coming years. However, the successful implementation of telemedicine relies on the willingness of both physicians and patients to embrace this digital healthcare feature, as well as on the government and healthcare institutions. This study serves as an initial step in gaining a deeper comprehension of the factors that encourage or impede Romanian physicians from adopting telemedicine services. Our study not only aims to uncover the perceptions, motivations, and barriers specific to Romanian physicians but also seeks to compare our findings with those from similar or more developed countries. By doing so, we aim to gain a broader understanding of the factors that truly influence the acceptance of telemedicine among physicians.

## Figures and Tables

**Table 1:** Descriptive statistics.

	Participants in Current Study N = 1093 (%)
**Sex**	
Male	334 (30.6%)
Female	759 (69.4%)
**Age**	
18–29 years old	14 (1.3%)
30–39 years old	311 (28.5%)
40–49 years old	365 (33.4%)
50–59 years old	274 (25.1%)
60–69 years old	119 (10.9%)
70–79 years old	10 (0.9%)
Over 80 years old	None
**Provenience**	
Rural	113 (10.3%)
Urban	980 (89.7%)
**Education**	
Medical school	161 (14.7%)
Medical Residency	343 (31.4%)
Ph.D. in medicine	285 (26.1%)
Post-university studies	282 (25.8%)
**Income**	
Under RON 4000 (EUR 806)	2 (0.2%)
RON 4000–6000 (EUR 807–1208 euro)	48 (4.4%)
RON 6001–8000 (EUR 1209–1610)	100 (9.1%)
RON 8001–10,000 (EUR 1611–2013)	172 (15.7%)
RON 10,001–12,000 (EUR 2014–2416)	234 (21.4%)
RON 12,001–14,000 (EUR 2417–2819)	161 (14.7%)
RON 14,001–16,000 (EUR 2820–3221)	149 (13.6%)
Above RON 16,000 (EUR 3221)	212 (19.4%)
Refuse to answer	15 (1.4%)
**Specialization**	
Resident physician	21 (1.9%)
Specialist physician	315 (28.8%)
Primary care physician	622 (56.9%)
Doctor in medical sciences	135 (12.4%)

**Table 2:** The reliability of measurement.

Variable	Composite Reliability	Cronbach’s Alpha	Average Variance Extracted (AVE)
Behavioral Intention	0.967	0.954	0.879
Perceived Usefulness	0.946	0.923	0.814
Perceived Ease of Use	0.918	0.864	0.788
Computer Self-Efficacy	0.900	0.851	0.694
Perceptions of External Control	0.954	0.927	0.873
Accessibility of Medical Records	0.955	0.929	0.875
Accessibility of Patients	0.971	0.955	0.917
Perceived Incentives	0.866	0.768	0.683
Computer Anxiety	0.951	0.922	0.865
Subjective Norms	0.920	0.883	0.741

**Table 3:** Square roots of AVE.

	BI	PU	PEOU	CSE	PEC	AMR	AP	PI	CANX	SN
**BI**	0.937	0.802	0.745	0.614	0.729	0.802	0.819	0.600	−0.229	0.780
**PU**	0.802	0.902	0.739	0.547	0.721	0.750	0.716	0.481	−0.158	0.729
**PEOU**	0.745	0.739	0.888	0.703	0.814	0.695	0.720	0.517	−0.283	0.629
**CSE**	0.614	0.547	0.703	0.833	0.637	0.555	0.601	0.469	−0.188	0.548
**PEC**	0.729	0.721	0.814	0.637	0.934	0.724	0.702	0.480	−0.286	0.617
**AMR**	0.802	0.750	0.695	0.555	0.724	0.935	0.726	0.489	−0.204	0.676
**AP**	0.819	0.716	0.720	0.601	0.702	0.726	0.958	0.533	−0.255	0.660
**PI**	0.600	0.481	0.517	0.469	0.480	0.489	0.533	0.827	−0.177	0.484
**CANX**	−0.229	−0.158	−0.283	−0.188	−0.286	−0.204	−0.225	−0.177	0.930	−0.108
**SN**	0.780	0.729	0.629	0.548	0.617	0.676	0.660	0.484	−0.108	0.861

## Data Availability

The data presented in this study are available on request from the corresponding author.

## Appendix A

**Table A1:** Path Coefficients of the Structural Model.

Estimated Coef.	Direct Effects			Indirect Effects		Total Effects		
Model	BI	PU	PEOU	BI	PU	BI	PU	PEOU
**BI**	-	-		-	-	-	-	-
**PU**	0.179 *** (*p* < 0.001)	-		-	-	0.179 *** (*p* < 0.001)	-	-
**PEOU**	0.047 (*p* = 0.059)	0.283 *** (*p* < 0.001)		0.051 ** (*p* = 0.009)	-	0.098 *** (*p* < 0.001)	0.283 *** (*p* < 0.001)	-
**CSE**	-	-	0.280 *** (*p* < 0.001)	0.013 (*p* = 0.268)	0.079 *** (*p* < 0.001)	0.027 (*p* = 0.100)	0.079 *** (*p* < 0.001)	0.280 *** (*p* < 0.001)
**PEC**	-	-	0.613 *** (*p* < 0.001)	0.029 (*p* = 0.088)	0.173 *** (*p* < 0.001)	0.060 ** (*p* = 0.002)	0.173 *** (*p* < 0.001)	0.613 *** (*p* < 0.001)
**AMR**	0.209 *** (*p* < 0.001)	0.255 *** (*p* < 0.001)		0.046 * (*p* = 0.016)		0.254 *** (*p* < 0.001)	0.255 *** (*p* < 0.001)	
**AP**	0.252 *** (*p* < 0.001)	0.142 *** (*p* < 0.001)		0.025 (*p* = 0.117)		0.278 *** (*p* < 0.001)	0.142 *** (*p* < 0.001)	
**PI**	0.120 *** (*p* < 0.001)	-				0.120 *** (*p* < 0.001)		
**CANX**	-	-	−0.078 ** (*p* = 0.005)	−0.004 (*p* = 0.432)	−0.022 (*p* = 0.151)	−0.008 (*p* = 0.361)	−0.022 (*p* = 0.151)	−0.078 ** (*p* = 0.005)
**SN**	0.252 *** (*p* < 0.001)	0.287 *** (*p* < 0.001)		0.051 ** (*p* = 0.008)		0.303 *** (*p* < 0.001)	0.287 *** (*p* < 0.001)	
**Age**	0.007 (*p* = 0.406)	-				0.007 (*p* = 0.406)		
**Gender**	−0.005 (*p* = 0.430)	-				−0.005 (*p* = 0.430)		
**Telemed**	0.033 (*p* = 0.135)					0.033 (*p* = 0.135)		
**R2/** **Adjusted R2**	84.6%/ 84.4%	71.5%/ 71.4%	72.7%/ 72.6%					
**Tenehaus GoF**	0.821 (large)							

*** *p*-value < 0.001; ** *p*-value < 0.01; * *p*-value < 0.05.

## Appendix B

**Table A2:** Effect Sizes for Direct, Indirect, and Total Effects.

Estimated Coef.	Direct Effects			Indirect Effects		Total Effects		
Model	BI	PU	PEOU	BI	PU	BI	PU	PEOU
**BI**	-	-	-	-	-	-	-	-
**PU**	0.146	-	-	-	-	0.146	-	-
**PEOU**	0.035	0.211	-	0.038	-	0.073	0.211	-
**CSE**	-	-	0.198	0.008	0.043	0.017	0.043	0.198
**PEC**	-	-	0.505	0.021	0.125	0.044	0.125	0.505
**AMR**	0.168	0.191	-	0.037	-	0.204	0.191	-
**AP**	0.207	0.103	-	0.021	-	0.228	0.103	-
**PI**	0.073	-	-	-	-	0.073	-	-
**CANX**	-	-	0.024	0.001	0.003	0.002	0.003	0.024
**SN**	0.202	0.211	-	0.041	-	0.243	0.211	-
**Age**	0.000	-	-	-	-	0.000	-	-
**Gender**	0.000	-	-	-	-	0.000	-	-
**Telemed**	0.009	-	-	-	-	0.009	-	-

## Appendix C

**Table A3:** The Questionnaire.

Construct	Items	
**Behavioral** **Intention**	BI1. I have a positive intention to adopt the telemedicine service. BI2. I will care for my patients through telemedicine service. BI3. I will gain accurate patient information and treatment histories. BI4. I will provide telemedicine services and share the information through this service.	[51]
**Perceived** **Usefulness**	PU1. It will positively affect the treatment plan. PU2. It is possible to provide a more comprehensive care service. PU3. It is efficient for diagnosing patients and scheduling. PU4. I can precisely monitor the patient’s condition.	
**Perceived Ease of Use**	PEOU1. It is easy to use the device for telemedicine service. PEOU2. It is easy to learn how to use the new device for telemedicine. PEOU3. It is easy to perform my job by using the telemedicine service.	
**Computer Self−Efficacy**	I could complete the job using a software package. CSE1. if there was no one around to tell me what to do as I go. CSE2. if I had just the built−in help facility for assistance. CSE3. if someone showed me how to do it first. CSE4. if I had used similar packages before this one to do the same job	[33]
**Perceptions of External** **Control**	PEC1. I have control over using the system. PEC2. I have the resources necessary to use the system. PEC3. Given the resources. opportunities. and knowledge it takes to use the system. it would be easy for me to use the system. PEC4. The system is not compatible with other systems I use.	
**Accessibility to Medical Records**	AMR1. I can gather correct information about the patient. AMR2. I can easily record a patient’s health condition. AMR3. Because of the precise record of the patients. it enables me to provide proper healthcare service to my patients.	[51]
**Accessibility to Patients**	AP1. I am able to care for patients living at further distances. AP2. I am able to be in contact with patients who seldom come to the clinic. AP3. I am able to be in contact with patients who cannot be easily delivered to the clinic.	
**Perceived** **Incentives**	PI1. This service needs proper government policy and support. PI2. It needs monetary incentives. PI3. It would be meaningful. if financial support were given.	
**Computer** **Anxiety**	CANX1. Computers do not scare me at all. CANX2. Working with a computer makes me nervous. CANX3. Computers make me feel uncomfortable. CANX4. Computers make me feel uneasy.	[33]
**Subjective Norms**	SN1.People who influence my behavior think that I should use the system. SN2. People who are important to me think that I should use the system. SN3. The senior management of this business has been helpful in the use of the system. SN4. In general. the organization has supported the use of the system.	

## Appendix D

**Table A4:** The Combined Loadings and Cross-Loadings of the Indicator Items.

	BI	PU	PEOU	CSE	PEC	AMR	AP	PI	CANX	SN
**BI_1_**	0.950	0.008	0.042	0.017	0.011	−0.169	−0.037	0.020	−0.007	0.019
**BI_2_**	0.955	−0.064	0.026	0.000	0.067	−0.196	0.072	−0.022	0.005	0.030
**BI_3_**	0.892	0.043	−0.110	0.040	−0.089	0.469	−0.150	0.039	−0.010	−0.056
**BI_4_**	0.952	0.016	0.035	−0.055	0.005	−0.074	0.106	−0.035	0.010	0.004
**PU_1_**	0.044	0.927	0.129	−0.009	−0.086	−0.049	−0.009	−0.005	0.006	0.003
**PU_2_**	0.085	0.931	−0.022	0.010	−0.091	−0.024	−0.056	−0.022	−0.010	−0.023
**PU_3_**	−0.130	0.881	0.024	−0.019	−0.012	0.065	−0.060	0.081	−0.002	0.045
**PU_4_**	−0.006	0.868	−0.138	0.018	0.203	0.012	0.131	−0.053	0.006	−0.024
**PEOU_1_**	−0.084	−0.190	0.931	0.008	0.079	0.048	0.009	0.002	−0.012	−0.008
**PEOU_2_**	−0.130	−0.244	0.914	0.037	0.041	−0.006	0.067	0.089	−0.031	−0.044
**PEOU_3_**	0.242	0.491	0.814	−0.050	−0.136	−0.047	−0.086	−0.102	0.048	0.060
**CSE_1_**	0.034	−0.174	0.427	0.753	0.330	−0.140	0.037	−0.133	−0.045	0.063
**CSE_2_**	−0.029	0.007	0.134	0.888	0.027	−0.083	0.061	−0.046	−0.016	0.020
**CSE_3_**	0.047	0.027	−0.204	0.869	−0.172	0.112	0.002	0.058	0.037	−0.078
**CSE_4_**	−0.051	0.124	−0.324	0.815	−0.151	0.100	−0.102	0.111	0.020	0.003
**PEC_1_**	0.012	0.094	0.067	0.045	0.926	−0.010	−0.027	−0.010	−0.006	−0.004
**PEC_2_**	−0.005	−0.053	−0.149	−0.059	0.937	0.007	0.024	−0.023	0.035	0.012
**PEC_3_**	−0.006	−0.040	0.083	0.014	0.940	0.004	0.003	0.033	−0.029	−0.009
**AMR_1_**	−0.010	0.012	−0.044	0.041	−0.037	0.934	−0.075	−0.005	0.030	−0.036
**AMR_2_**	−0.073	−0.134	0.072	−0.012	0.075	0.938	0.067	−0.010	−0.036	0.036
**AMR_3_**	0.084	0.123	−0.028	−0.028	−0.039	0.935	0.007	0.016	0.006	0.000
**AP_1_**	0.014	0.127	−0.055	0.005	−0.011	0.003	0.942	−0.019	0.020	0.006
**AP_2_**	−0.035	−0.064	0.026	−0.006	0.000	0.031	0.968	0.005	−0.015	0.021
**AP_3_**	0.022	−0.060	0.028	0.002	0.010	−0.034	0.962	0.013	−0.005	−0.026
**PI_1_**	−0.105	−0.243	0.227	0.057	−0.017	0.015	0.086	0.804	−0.067	−0.047
**PI_2_**	−0.379	0.096	−0.207	−0.004	0.060	0.025	0.043	0.838	0.037	0.062
**PI_3_**	0.480	0.137	−0.011	−0.050	−0.044	−0.039	−0.126	0.837	0.027	−0.017
**CANX_2_**	0.037	−0.020	−0.050	−0.029	−0.017	0.006	0.012	0.000	0.905	0.037
**CANX_3_**	−0.029	0.005	0.041	0.011	−0.005	0.000	0.012	0.025	0.939	−0.024
**CANX_4_**	−0.007	0.014	0.007	0.016	0.021	−0.005	−0.023	−0.025	0.947	−0.012
**SN_1_**	−0.665	0.028	−0.198	0.003	0.217	0.091	0.061	0.000	0.014	0.812
**SN_2_**	−0.478	0.042	−0.072	−0.054	0.128	0.129	0.040	−0.005	0.010	0.879
**SN_3_**	0.445	−0.015	0.099	0.022	−0.153	−0.115	−0.008	0.018	0.000	0.879
**SN_4_**	0.653	−0.053	0.158	0.029	−0.176	−0.100	−0.089	−0.013	−0.023	0.871
